# The covid-19 pandemic and international mobility in Brazil: challenges for the health and social protection of international migrants in times of uncertainty

**DOI:** 10.1590/S0104-59702023000100033

**Published:** 2023-08-14

**Authors:** Daniel Granada, Cássio Silveira, Silvia Regina Viodres Inoue, Regina Yoshie Matsue, Denise Martin

**Affiliations:** i Professor, Departamento de Ciências Naturais e Sociais/Universidade Federal de Santa Catarina. Curitibanos – SC – Brasil daniel.granada@ufsc.br; ii Professor, Faculdade de Ciências Médicas da Santa Casa de São Paulo/Universidade Federal de São Paulo. São Paulo – SP – Brasil cassiosilveirasc@gmail.com; iii Pesquisadora independente, Grupo de Pesquisa “Processos Migratórios Internacionais e Saúde Coletiva”. São Paulo – SP – Brasil silviaviodres@gmail.com; iv Professora, Escola Paulista de Medicina/Universidade Federal de São Paulo.São Paulo – SP – Brasil rymatsue08@unifesp.br; v Professora, Escola Paulista de Medicina/Universidade Federal de São Paulo. São Paulo – SP – Brasil denise.martin@unifesp.br

**Keywords:** Pandemic, Covid-19, Health and human mobility, Brazil, Nationalism, Pandemias, Covid-19, Saúde e mobilidade humana, Brasil, Nacionalismo.

## Abstract

The relationship between health and migration was severely affected during the covid-19 pandemic. This article discusses the literature on health and migration, including the deteriorating levels of social and economic vulnerability of international migrants to Brazil in 2020 and 2021. The analysis was based on a review of articles published in the national and international press addressing the impacts of covid-19 on contemporary processes of human mobility and its consequences for stigmatized populations. The results point to the possibility of understanding the pandemic as a key moment for rethinking nationality and borders.

## Resumo

As relações entre saúde e migrações foram duramente afetadas durante a pandemia de covid-19. O presente artigo discute a produção sobre saúde e migrações e problematiza o agravamento da condição de vulnerabilidade social e econômica dos migrantes internacionais durante 2020 e 2021, no Brasil. A pesquisa vem sendo realizada por meio de revisão da literatura de matérias retiradas da imprensa nacional e internacional e busca abordar os impactos da covid-19 nos processos contemporâneos de mobilidade humana e quais suas consequências para as populações estigmatizadas. Os resultados apontam para a possibilidade de se compreender a pandemia como um momento-chave para repensar nacionalidade e fronteira.

Existem diversas formas de contar o desenrolar de uma pandemia: podemos contabilizar a evolução do número de mortos, elencar as medidas colocadas em prática por um determinado governo para conter – ou não – a propagação do contágio entre a população, podemos ainda calcular o volume de dinheiro despendido em estratégias mais ou menos eficazes para a contenção do vírus. No Brasil, contabilizamos em números oficiais mais de 668 mil mortos (Brasil, 2019). Dificilmente esses números podem fornecer a exata dimensão humana do sofrimento das pessoas que perderam seus entes queridos, do sofrimento e desolação daqueles que precisaram de ajuda e encontraram unidades de saúde e hospitais lotados, ou, ainda, da carência de quem estava afastado do seu país de origem e precisou atravessar esse momento delicado longe de seus familiares. Este artigo analisa desafios enfrentados pelas pessoas em mobilidade internacional durante a pandemia de covid-19 no Brasil e busca trazer à luz algumas contribuições para pensar sobre as relações entre saúde e mobilidade humana no contexto da maior crise sanitária experimentada até então pela nossa geração. Trata-se de reflexões preliminares que têm por objetivo elaborar um quadro visando a uma melhor abordagem do fenômeno, relacionando saúde e mobilidade humana no contexto da pandemia.

Até 2020 em todo o mundo 82,4 milhões de pessoas foram obrigadas a se deslocar em razão de perseguição, conflito, violência, violação de direitos ou eventos que perturbam seriamente a ordem pública (Acnur, 2022). Muitas dessas pessoas padecem diante de muros, desertos, mares, campos de refugiados e outras técnicas de fronteirização nas regiões limítrofes, que as colocam em permanente quarentena e perigo nos campos de refugiados, rompendo barreiras por destinos incertos e condições de vida insalubres nos países receptores, expostos aos mais diversos problemas sanitários, condições deletérias de trabalho, insegurança alimentar e habitacional, entre outros. De maneira geral, os processos sociais e os contextos sociopolíticos quase sempre são desfavoráveis à saúde daqueles que migram (Goldberg, Martin, Silveira, 2015).

A pesquisa, nesta fase inicial, tem sido realizada por meio de revisão da literatura sobre saúde e migrações, bem como sobre os impactos da pandemia nas populações em situação de mobilidade internacional. Também temos utilizado o arquivo de notícias relacionadas ao tema que foi publicado na imprensa internacional e nacional durante o período da pandemia, com foco nas questões de saúde e mobilidade humana, questões que a pandemia veio notavelmente colocar à tona, fato que os pesquisadores que têm se dedicado há alguns anos à abordagem sobre saúde e migrações têm pontuado e que emergem de modo evidente em diversos trabalhos aqui discutidos. Se, por um lado, a pandemia colocou em relevo de maneira inegável as relações entre saúde e migrações e diversos colegas que há tempos estudam migrações empreenderam esforços no sentido de redirecionar seus temas de pesquisa para dar conta do fenômeno da pandemia e seu impacto sobre as populações migrantes, por outro lado, os autores que há anos se dedicam ao tema vêm problematizando essas relações, demonstrando como a saúde e as migrações são temas de pesquisa que fazem parte da própria formação do Estado-nação no Brasil (Granada et al., 2017).

Com este artigo pretendemos ampliar o debate sobre algumas das consequências sofridas pelos migrantes internacionais no Brasil no contexto da pandemia de covid-19. Para tanto, procuramos relacionar os deslocamentos migratórios internacionais no Brasil com os seguintes aspectos: os processos de vulneração acentuados pelas desigualdades estruturais, sobretudo entre as mulheres migrantes; o tortuoso circuito de informações sobre a doença (sintomatologia e prevenção); a inexistência e/ou impossibilidade de acesso a orientações e isolamento; as intercorrências xenofóbicas das associações entre migrantes e patologias; assim como os riscos vivenciados no trabalho e, muitas vezes, potencializados pela ausência do acesso aos serviços de saúde.

## Migrações no Brasil: uma breve contextualização

As relações entre saúde e mobilidade humana no Brasil são históricas e complexas. Primeiramente, é importante assinalar que o Brasil recebeu migrantes voluntários e, principalmente, forçados desde o século XVI. Ainda durante o século XIX permaneciam as migrações forçadas via o tráfico de povos africanos, simultaneamente à vinda de imigrantes europeus desde o início desse período. Entretanto, o tipo e o perfil do imigrante mudaram ao longo do tempo (Seyferth, 1996). A chegada mais intensa de imigrantes no país é registrada no final do século XIX até a década de 1920, quando chegaram principalmente portugueses, italianos e espanhóis que vinham para realizar a substituição da mão de obra escrava para o trabalho livre nas lavouras da jovem república brasileira (Schwarcz, 1995; Seyferth, 2000; Skidmore, 1989). As ideias sobre o branqueamento da população brasileira com o intuito de inserir o Brasil em um projeto de país viável por meio da "melhoria da raça", proporcionada pelo "caldeamento" das populações locais com populações brancas de origem europeia (Schwarcz, 1995; Chalhoub, 1993; Seyferth, 1996; 2000; Skidmore, 1989), também podem ser compreendidas dentro das relações entre saúde e mobilidade humana.

A mobilidade humana internacional está intrinsecamente associada, ao longo da história do Brasil, à disseminação das epidemias. Já em 1850, o movimento antiescravista imputava a disseminação da febre amarela no Brasil à vinda dos escravos africanos (Kodama, 2009). A incidência de determinados tipos de doenças sobre alguns grupos populacionais, como a tuberculose e a varíola que atingiam preferencialmente os negros, comparativamente a doenças que vitimavam mais diretamente os imigrantes recém-instalados no Brasil, como no caso da febre amarela, levavam o governo a ter maior ação destinada ao contingenciamento das doenças que tinham maior letalidade sobre os imigrantes do que aquelas que atingiam mais a população negra (Chalhoub, 1993).

No caso da peste bubônica, entre 1889 e 1904, as políticas de imposição de quarentenas, bem como restrições de chegada de navios oriundos de determinados portos (Nascimento, Silva, 2013; Silva, 2015) também demonstram o impacto da mobilidade humana na disseminação do contágio e a necessidade de controle das doenças por meio de medidas de restrição da mobilidade. Do mesmo modo, como ocorreu no caso da "peste branca" ou tuberculose na virada do século retrasado, a disseminação dessas doenças, que afetavam especialmente as zonas portuárias das cidades, tinha efeito dissuasivo para a chegada dos imigrantes europeus (Bertolli-Filho, 2001).

A formação populacional no Brasil se relaciona, portanto, de forma mais ou menos intensa ao processo de chegada desses fluxos de imigrantes ao longo do século XX, imigrantes esses que viam no país a possibilidade de construir novos projetos de vida.

Durante os anos 1980, o Brasil vai assistir pela primeira vez ao processo de emigração de brasileiros. Em um contexto de degradação das condições de vida, relacionada com a chamada "década perdida para o desenvolvimento", milhares de brasileiros partem na esperança de encontrar melhores condições de vida e trabalho nos países do norte e em busca da ascensão social que lhes era negada no país (Margolis, 1994, 2003; Martes, 1999, 2003; Martes, Fleisher, 2003; Patarra, 2005; Reis, Sales, 1999; Ribeiro, 1999).

No passado próximo, com a estabilidade econômica que havia sido conquistada no início do século XXI, com mais evidência no cenário internacional e um processo de expansão da economia, o Brasil tornou-se novamente um país atraente para os estrangeiros e brasileiros retornados. Somado a isso, deve-se considerar o movimento de brasileiros que, principalmente em razão da crise econômica, retornam a partir de 2007 dos EUA e Europa. Esses fluxos são compostos por estudantes e trabalhadores altamente qualificados, refugiados políticos, sobreviventes de catástrofes ambientais, entre outros. Com relação aos motivos dos deslocamentos, devem ser levados em conta fatores como reagrupamento familiar, refúgio e asilo, além de questões laborais (Cavalcanti, Oliveira, Tonhati, 2015).

Depois de 2015, porém, a crise econômica e a ascensão da extrema direita ao poder modificaram esse cenário, afetando diretamente os fluxos. A degradação das condições de vida, associada ao desemprego, à inflação e à falta de perspectivas com relação ao futuro levaram novamente o Brasil a se tornar um país de emigração. Assim como na chamada década perdida para o desenvolvimento, de 1980 a 1990, o período de 2015 a 2021, quando estamos em plena pandemia, passou a ser o período de maior êxodo de brasileiros do país, tendo o número de brasileiros vivendo no exterior saltado de 1,9 milhão em 2012 para 4,9 milhões em 2021 (Veiga, 13 dez. 2021).

Concomitantemente, nas últimas décadas, o Brasil tornou-se o destino de muitos migrantes, principalmente originários de países da América do Sul e Central: Bolívia, Peru, Haiti, Venezuela, e de países africanos: Congo, Senegal, entre outros. De acordo com Baeninger et al. (2021), o Sistema Nacional Migratório (Sismigra), do Ministério da Justiça/ Polícia Federal, registrou, entre 2000 e março de 2020, que 1.504.736 pessoas entraram no país, oriundas de 227 localidades diferentes, sendo a maioria delas do sul global (Baeninger et al., 2021).

É preciso colocar em relevo a aprovação do novo marco legal das migrações no país. A lei n.13.445/17 (Lei de Migração), aprovada em 24 de maio de 2017, buscou oferecer garantia dos direitos dos imigrantes e emigrantes. A nova lei vem ao encontro da mobilização de acadêmicos e diversos atores da sociedade civil que clamavam pela substituição do Estatuto do Estrangeiro, elaborado durante a ditadura militar e que ficou em vigor entre 1980 e 2017. Esse estatuto era entendido como obsoleto, pois percebia o imigrante e a imigração como potencial ameaça à segurança do país. O novo marco legal sobre as migrações no país pode ser considerado uma das legislações mais vanguardistas da região com relação à proteção migratória (Cavalcanti, Oliveira, Silva, 2020).

Durante a pandemia de covid-19, a mobilidade humana internacional foi duramente afetada. No Brasil não foi diferente. O fechamento de fronteiras, as medidas restritivas de contato e a política de concessão de vistos (Cavalcanti, Oliveira, Silva, 2020) provocaram a interrupção ou postergação dos projetos migratórios. O fechamento das fronteiras por vias terrestres, aéreas, marítimas e fluviais tornou-se uma medida corrente para o contingenciamento da disseminação do vírus, o que reduziu de modo significativo a mobilidade internacional. Por meio da análise das portarias interministeriais publicadas pelo governo brasileiro, Ribeiro e Sena (2021) afirmam que fica patente que a entrada de venezuelanos e venezuelanas foi o principal objeto de restrição, o que, segundo eles, configuraria uma seletividade migratória a partir da determinação de quem pode ou não entrar no país.

Esse cenário demonstra a complexidade dos fluxos migratórios na atualidade. Por um lado, continua ocorrendo a emigração de brasileiros de forma intensa, especialmente do fenômeno chamado fuga de cérebros, ao mesmo tempo que o país atrai novos e diversificados fluxos de imigrantes (Silveira, 18 jan. 2020). Os novos fluxos de imigração para o Brasil colocam em destaque o debate sobre direitos humanos, a garantia de acesso aos serviços de saúde, bem como a absorção dessa população a postos de trabalho dignos no mercado de trabalho local e sua consequente integração na sociedade. A complexidade dos fluxos migratórios, de e para o Brasil, se relaciona com o contexto de um país de mudanças políticas e sociais abruptas e que impactam de modo profundo o contexto econômico e, por consequência, as oportunidades de trabalho e as condições de vida de imigrantes e não imigrantes. Portanto, ao analisar os fluxos migratórios e a mobilidade humana internacional, devemos estar atentos à diversidade e à complexidade do fenômeno. A produção acadêmica no âmbito das discussões sobre as migrações internacionais também tem acompanhado esse crescimento, e diversos aspectos têm sido abordados nos estudos relacionados às temáticas de imigração e emigração no Brasil (Ferreira, Povoa Neto, Santos, 2010; Silva, 2012; Povoa Neto, Santos, 2013; Cavalcanti, Oliveira, Tonhati, 2015). Apesar dessa crescente produção, ainda são necessários estudos que analisem os efeitos da imigração, seja na saúde ou no trabalho, e suas interfaces com questões de gênero e direitos humanos, bem como enfocando as desigualdades sociais no período da pandemia.

## O contexto da pandemia e os migrantes internacionais

As medidas de prevenção e combate ao contágio e disseminação do coronavírus tornaram-se um campo de disputa e se constituíram em verdadeira arena de debates político-ideológicos no Brasil. Durante 2020 e 2021, houve um contexto de muita confusão, disseminação de notícias falsas, ataques à ciência e ao conhecimento científico de modo muito marcante no país. Esses fatores exigiram grande mobilização local dos municípios na observação das restrições às aglomerações, às condições de mobilidade e circulação das pessoas. Tais medidas demandam esforços individuais e coletivos que são condicionados pela situação socioeconômica, de moradia, de acesso à saúde e à educação, entre outras, de cada pessoa e/ou grupo social e, apesar de necessárias, produziram grandes impactos socioeconômicos na maior parte da população. Tais impactos ocorrem de maneira desigual, afetando fundamentalmente as populações que vivem, precariamente, à margem da sociedade (Barberia, Costa, Sabino, 2021), dentre elas, migrantes internacionais e refugiados (Baeninger et al., 2021).

Nesse sentido, a pandemia exacerbou as desigualdades sociais estruturais presentes em nossa sociedade, deixando as populações mais pobres e vulneráveis entregues aos mais diferentes tipos de riscos e ameaças. Considerando que a pandemia não se constitui em um fenômeno uniforme, ou seja, ela atinge de forma desigual diferentes grupos na sociedade, existe a necessidade de compreender seus impactos em grupos já considerados vulneráveis, como a maior parte das pessoas em situação de mobilidade internacional. Em diversos países e no Brasil, a população em deslocamento, migrante ou refugiada, é um dos grupos que foram severamente atingidos pela crise. A mobilidade humana é um fenômeno global que perpassa a história da humanidade, precedendo, até, a existência dos Estados nacionais. Contudo, na atualidade presenciamos uma crise humanitária sem precedentes em torno dos deslocamentos em massa oriundos de vários países do sul (Magalhães, Bogus, Baeninger, 2018), seja em razão de conflitos internos, desastres ambientais, insegurança material e física, intolerância, exclusão econômica e política, repressão e transtornos diversos (Acnur, 2022). Com o surgimento da pandemia, assistimos a políticas de restrição de mobilidade aplicadas como medidas sanitárias, além do chamado "distanciamento físico" ou "distanciamento social" imposto em diversos países, também houve um endurecimento de fronteiras, com o impedimento ou limitação da circulação internacional de indivíduos (Aquino et al., 2020).[1] Com relação ao distanciamento físico, muitos migrantes tinham dificuldades estruturais e materiais para adotá-lo, fosse em razão da moradia em residências coletivas, nas quais obedecer às regras de distância dos outros moradores era tarefa árdua, senão impossível, fosse porque estavam empregados nos setores considerados "serviços essenciais" e, portanto, não puderam parar de trabalhar, ou, em situação ainda mais dramática, aqueles que não conseguiram o auxílio emergencial por razões diversas agravadas pelo desconhecimento dos próprios direitos, desinformação sobre a disponibilidade desse recurso, dificuldade com o idioma local, documentação irregular, entre outras (Villalón, Moya, Bejarano, 2021).

Com relação às restrições de circulação entre as fronteiras nacionais, em alguns casos, a imposição de restrições passou a ser associada a determinada nacionalidade do país de origem, trazendo à tona o debate sobre os perigos iminentes de xenofobia, uma vez que se associa a doença ao corpo do imigrante de determinada nacionalidade (Fassin, 2001).

## Vírus e mutações têm nacionalidade?

No início da pandemia no Brasil, houve, por parte de algumas personalidades políticas, a tentativa de associação do vírus com a China. Não apenas por aqui, outros países também designavam a pandemia associada a uma suposta origem nacional, o "vírus chinês". Na França, por exemplo, houve diversos casos de jornais que colocaram na capa a expressão "vírus chinês" para alertar sobre a pandemia logo no seu início (Maussion, 23 jan. 2020). Uma pesquisa realizada por meio da análise do fluxo de mensagens no Twitter (Budhwani, Sun, 2020) com os descritores "China vírus" ou "*chinese virus*" concluía que o aumento das referências a esses descritores na rede social estava ocorrendo e se disseminando *on-line* e que o estigma associado à covid-19 poderia ser perpetuado (Hillard, 28 jan. 2020).

Chan e Strabucchi (2021) mostram como a pandemia afetou as posições sociais de grupos étnicos chineses (*ethnic chinese*) e populações do Leste Asiático que vivem no Chile. Para as autoras, a pandemia provocou o ressurgimento de discursos racistas antigos sobre esses grupos, considerados atrasados, associados à sujeira e à doença, ou seja, não pertencentes à "democracia e liberdade chilenas".

No sentido contrário, o exemplo do Peru demonstra que as manifestações nacionalistas, o racismo e a xenofobia relacionadas aos chineses, muito comuns em dois séculos de história naquele país, não emergiram durante a pandemia da covid-19. Apesar da forte expressão de uma opinião pública que responsabilizou os trabalhadores informais (nacionais e estrangeiros) que representavam cerca 72% da força de trabalho, o país foi envolvido por medidas sanitárias precocemente implementadas, como a quarentena, por exemplo, e promoveu articulações internacionais com a República Popular da China, diferentemente dos demais países da região da América do Sul. Com isso, uma nova configuração geopolítica emergiu dessa articulação binacional, que incluiu remessas de insumos para a saúde pública peruana, particularmente a vacina desenvolvida pelos chineses (Ragas, Palma, 2022).

O emprego da expressão "vírus chinês" foi rechaçado oficialmente pela Organização Mundial da Saúde (OMS) logo no início da pandemia. No Brasil, algumas vozes também se levantaram contra o racismo e a xenofobia que eram provocados pela designação de origem do vírus. A professora Deisy Ventura (11 set. 2020), em artigo publicado no site do Museu da Imigração de São Paulo, alertava para os riscos de a pandemia servir como salvo-conduto para o racismo e a xenofobia, afirmando que caberia ao Estado e à sociedade o combate ao estigma associado à covid-19 em todas as suas formas.

Os ecos dessa polifonia de vozes nos webinários, *lives*, publicações em mídias sociais e matérias jornalísticas conferiram visibilidade às migrações e à mobilidade no contexto da covid-19. Por um lado, incrementaram as iniciativas fundamentais da própria sociedade civil envolvendo migrantes e não migrantes para o enfrentamento à covid-19 e suas repercussões com provisão material de alimentos e itens de necessidade básica, produção e divulgação de informações sobre saúde, legislação vigente sobre aluguéis, despejo e benefícios. Por outro lado, ecoavam vozes xenofóbicas. Em análise de publicações em um dos jornais de maior circulação no Brasil, a pesquisadora Julia Alves Brasil (2021) constatou que a familiarização desse novo objeto social fez com que estereótipos negativos fossem reforçados. A migração foi frequentemente representada como uma questão política com aspectos econômicos e de segurança, e a intensificação desses estereótipos negativos relacionados aos migrantes levou à ocorrência de novos episódios de xenofobia e ampliação da securitização das migrações.

Se no início da pandemia causava preocupação associar um vírus a uma determinada nacionalidade de origem, parece que com seu desenrolar a atribuição de determinadas nacionalidades a suas variantes se tornou prática corrente. Entre 2020 e 2021, falava-se abertamente na imprensa sobre a "variante inglesa", a "variante sul-africana", e mais recentemente sobre a "variante brasileira", ou "amazônica" e a "variante indiana" do vírus. A própria OMS teria emitido um alerta em que expressava preocupação com a disseminação da "variante brasileira" (Mendes, 16 jan. 2021). As consequências relacionadas ao descontrole evidente da pandemia no país, bem como à descoberta da "variante brasileira", tiveram como efeito a criação de políticas de restrição de entrada baseadas na nacionalidade do país de origem. Em janeiro de 2021, mais de trinta países tinham restrições de viagens que impediam a entrada de brasileiros, alguns como os EUA

impediam a entrada de qualquer pessoa, independentemente da nacionalidade, tendo visitado o Brasil nos últimos 14 dias, e o Uruguai só aceitava a passagem de brasileiros em trânsito para outros países nos aeroportos.

Mais de dois anos depois do início da pandemia, novas restrições de mobilidade e fechamentos de fronteiras aconteceram. As justificativas foram as novas variantes do vírus e a baixa taxa de vacinação (ou hesitação vacinal) em alguns países. Pai e Olatunbosun-Alakija (2021) apontam que a pandemia já deixou 100 milhões de pessoas na pobreza e que as Nações Unidas estimam que os países em desenvolvimento sofrerão perdas econômicas de $12 trilhões até 2025. Na perspectiva desses autores, os líderes mundiais falharam em assegurar uma rápida e equilibrada distribuição de diagnósticos e vacinas, indo além dos nacionalismos. Em vez de isolar e discriminar, por que não se viabilizou a vacinação em massa para os países pobres?

Ao que parece, se no início da pandemia a associação à China era indesejada, em um segundo momento, com o surgimento das variantes do vírus, o preconceito com relação aos brasileiros em outros países em função da falta de controle da pandemia aumentou bastante, especialmente durante 2021, com a disseminação da "variante brasileira", o que tornou impossível o deslocamento de brasileiros durante um longo período para mais de trinta países. Se as fronteiras nacionais não dão mais conta de conter os vírus e as doenças que os migrantes supostamente carregam consigo para outros países, fica claro que a atribuição de uma nacionalidade a um vírus ou sua variante parece estar muito mais associada à importância econômica e à força política de determinado país no cenário internacional do que a outros fatores.

O Brasil, no cenário de descontrole da pandemia, estava atravessando uma crise econômica, social, política e humanitária sem precedentes, que ainda se arrasta, tornando-se um pária no cenário internacional. Ticktin (s.d.) assinala que a pandemia despertou a ideia equivocada e mortal de que as nações precisavam fechar as fronteiras contra a chegada dos "outros" invasores; afirma ainda que as "quarentenas nacionais" não fizeram sentido e que os Estados-nação claramente não são "espaços seguros". A autora também critica a imposição dos chamados "passaportes vacinais", alegando que o passaporte vacinal contém a ideia de tratar o corpo dos indivíduos, bem como a evidência biológica, como fonte primária de verdades sobre as pessoas (Ticktin, s.d.).

Segundo Cavalcanti e Oliveira (2020), as fronteiras do país viram a mobilidade de pessoas ser drasticamente afetada durante os primeiros seis meses da pandemia. Nas fronteiras brasileiras, em 2019, o volume médio mensal de entradas e saídas que era de aproximadamente 2,5 milhões reduziu-se para cerca de 90 mil nos meses de abril e maio de 2020 e ainda para menos de 40 mil em junho e julho. O fechamento de fronteiras brasileiras para entrada de estrangeiros representa um obstáculo, especialmente para pessoas refugiadas no processo de reunificação familiar (Martuscelli, 2020). Entretanto, esse fechamento não cessou o deslocamento de pessoas. Mesmo se o ingresso oficial foi drasticamente reduzido, criaram-se novas rotas obscuras e mais perigosas que as oficiais, aumentando assim a vulnerabilidade dos deslocamentos.

As ciências sociais e humanas utilizam o conceito de Estado-nação como unidade de análise. Wimmer e Glick-Schiller (2002) definem criticamente o nacionalismo metodológico como a certeza de que nação/Estado/sociedade é a forma política e social naturalmente concebida no mundo moderno. A crítica dos autores a esse conceito é que ele se fixa nas bordas territoriais de um Estado-nação e, também, na identificação com a construção de um projeto de Estado. Quando pensamos sobre a nacionalidade e questões de saúde e doença, frequentemente somos levados a indagar se a nacionalidade pode ter alguma importância enquanto um determinante social de saúde. A "nacionalidade", de modo semelhante à "raça", possui pouca ou quase nenhuma significação em termos biológicos, entretanto, da mesma forma que a cor da pele, em determinados contextos, a ideia de um pertencimento nacional, consubstanciado como uma essência, cristalizada na nacionalidade, pode induzir a processos de discriminação e xenofobia que podem vir a causar prejuízos e desvantagens às pessoas em situação de mobilidade internacional.

## Trabalho e vulnerabilidades de migrantes internacionais na pandemia

A crise sanitária decorrente da disseminação da covid-19 em escala global gerou impactos diversos nos processos de circulação e mobilidade internacional a partir do Brasil. O relatório anual do Observatório das Migrações de 2020 apresenta gráficos impressionantes, mostrando como as medidas restritivas de circulação impactaram de forma sem precedentes a entrada de imigrantes e refugiados no país (Cavalcanti, Oliveira, 2020).

Apesar da queda brusca nos números registrada no relatório do Observatório das Migrações de 2020, era possível ler no relatório de 2021 que os empregos para imigrantes não apenas resistiram à crise, mas variaram positivamente, apresentando aumento entre 2019 e 2020:

> Durante a pandemia de covid-19 os empregos para os imigrantes não somente resistiram à crise, bem como apresentaram uma variação positiva. Constatou-se um aumento de cerca de 23% entre os anos de 2019 e 2020. Esse fato pode ser explicado pelas particularidades dos nichos laborais, principalmente o final da cadeia produtiva do agronegócio, na empregabilidade dos imigrantes. Com um perfil eminentemente exportador, esse nicho de produção é mais resistente às crises econômicas devido a sua consolidada participação no mercado global. Durante a pandemia de covid-19, esse setor era considerado serviço essencial por ser produtor de alimentos (Cavalcanti, Oliveira, Silva, 2021, p.15-16).

É importante ponderar que não há nada para comemorar no fato de ter ocorrido uma variação positiva da empregabilidade dos imigrantes no período da pandemia. Pelo contrário, esse número também pode indicar que, em razão da própria condição de imigrante, essa categoria de trabalhadores, frequentemente mais vulnerável que as populações locais, não possuía outra opção senão a de aceitar as ofertas de trabalho, mesmo se expondo aos riscos de contágio, aceitando condições de trabalho precárias em plena pandemia, como é o caso dos trabalhadores migrantes em frigoríficos (Granada et al., 2021). Outro dado interessante que contribui com essa análise trata do aumento do número de contratos de trabalhos encerrados por morte, que subiu 33% no primeiro bimestre de 2021 em comparação com o mesmo período de 2020, com números expressivos no setor de serviços, indústria, comércio e construção civil. Nesses setores ditos essenciais, os trabalhadores não podem parar e, se pararem, podem ser substituídos a baixo custo por um exército de reserva gerado pela crise (Rodrigues, 8 abr. 2021).

Ressaltamos também que as vulnerabilidades se agravaram ainda mais entre as mulheres que são corpos duplamente dissidentes, como mulher e como migrante (Matsue, 2022). Além do risco de contágio, muitas delas enfrentaram diversas violências – no trabalho ou no ambiente doméstico (Marinucci, 2022). Geralmente, as mulheres migrantes atuam em ocupações consideradas a "linha de frente" na pandemia, sendo mais afetadas pelos impactos da covid-19 em saúde, precariedade nas condições de trabalho, e redução no ingresso ocupacional. No Brasil, o maior impacto laboral da covid-19 recaiu também sobre as mulheres migrantes (Tonhati, Macêdo, 2021). A análise de dados (de janeiro a setembro de 2019 e 2020) do Sistema de Tráfego Internacional de dados da Polícia Federal, do Sismigra e do Cadastro Geral de Empregados e Desempregados do Ministério da Economia constatou que, além da ruptura na tendência crescente de admissões no período de 2010 a 2019, as mulheres imigrantes perderam mais postos de trabalho formal, vivenciaram aumento da empregabilidade em frigoríficos e da exposição à contaminação pelo coronavírus nesses ambientes fechados (Tonhati, Macêdo, 2021). Quanto à maioria das migrantes, sem trabalho formal, a sobrevivência de suas famílias foi afetada pela interrupção obrigatória de atividades "não essenciais" e, consequentemente, pela perda de renda (Villalón, Moya, Bejarano, 2021).

Nesse cenário, milhares de migrantes vivem em condições precárias de moradia nas grandes cidades brasileiras: são moradores de cortiços, ocupações, oficinas de costura ou em centros de acolhida, além daqueles em situação de rua. São alvos de discriminação social e racial, e seus direitos, na maioria das vezes, não são respeitados nos espaços públicos, intensificando a sua condição de vulnerabilidade (Santos, 2015; Goldberg, Sow, 2017; Risson, Matsue, Lima, 2018). Além de outras morbidades presentes nessa população, de modos distintos essas pessoas estão sujeitas a espaços e circunstâncias que potencializam a contaminação por diversas patologias (Goldberg, Silveira, 2013; Silveira et al., 2016; Luna et al., 2017; Carneiro-Junior et al., 2018; Haydu et al., 2019). A maioria não tem opção de isolamento; por exemplo, a comunidade boliviana da região metropolitana de São Paulo que habita e trabalha em espaços fechados de oficinas de costura.

Em razão de uma condição de vida e saúde frequentemente mais vulnerável, em várias situações, em comparação com as populações locais, é importante entender como os efeitos da pandemia impactaram a vida de migrantes e pessoas refugiadas e de que forma vieram a potencializar a precariedade da vida e as situações de violência estrutural já bastante discutidas na literatura (Farmer, 2004). A paralisação das atividades laborais e a perda de postos de trabalho por grande parte dessas pessoas agravaram a instabilidade dos recursos e meios de sobrevivência, como a satisfação das necessidades básicas diárias das famílias e a manutenção da moradia, acentuando a insegurança alimentar, por exemplo, o impedimento de trabalho para os vendedores ambulantes e trabalhadores diaristas dos grandes centros urbanos.

A exposição da população vulnerável ao contágio pela covid-19 veio potencializar e tornar mais evidentes as desigualdades já existentes em nossa sociedade. Desse modo, milhões de brasileiros compartilham com os migrantes internacionais, ainda que com intensidades diferentes, o drama imposto pela disseminação do vírus e adoecimento da população. Assim, continuam sendo vítimas das políticas econômicas que concebem a desigualdade social como um valor positivo e, em consequência, tem provocado um número exacerbado e evitável de mortes em decorrência da covid-19. A regulação da vida foi transformada na regulação da morte em condições extremas produzidas pela sociedade. A morte ocorre pelo empobrecimento das minorias e pelo não direito à vida e à saúde; são corpos que já estavam expostos ao esgotamento físico e a substâncias tóxicas no trabalho, agora também expostos ao coronavírus (Mbembe, 2020).

Segundo Lima e Silva (2017), a Comissão Interamericana dos Direitos Humanos considera os migrantes mais vulneráveis do que os indivíduos locais de um estado ou país e recomenda medidas para a diminuição das desigualdades. No contexto da pandemia, o governo brasileiro aprovou o projeto de lei n.1066/2020 (Brasil, 2020), instituindo um benefício emergencial para famílias vulneráveis, mas resultou em muitas dificuldades de acesso a essa população. O acesso ao auxílio no Brasil atravessado por obstáculos como a falta de informação centralizada e a ausência de plataformas digitais inclusivas se tornou ainda menos tangível para os indocumentados ou para aqueles que chegaram há pouco tempo no país.

Nessa perspectiva, o Sistema Único de Saúde (SUS) se apresenta como uma importante política pública. Assentado sobre os princípios de universalidade, equidade e integralidade, dispõe que todos devem ter acesso aos cuidados em saúde por meio de sua rede de atenção à saúde, mesmo aqueles que não portam qualquer documento de identificação. Assim, garante-se atendimento à saúde a todas as pessoas a partir de sua chegada ao Brasil, sem qualquer ônus ou penalidade devido à sua condição (Brasil, 2017). Mais que isso, de acordo com o princípio da equidade, sua vulnerabilidade deve ser considerada e, portanto, o Estado deve tratar de forma diferente os indivíduos a fim de que cada um tenha as mesmas oportunidades, no sentido da igualdade e de justiça social, ou seja, deve levar em conta as diferenças sociais, biológicas, econômicas e culturais dos sujeitos (Lima, Silva, 2017; Paim, 2013; Martes, Faleiros, 2013).

O SUS, contudo, ainda enfrenta dificuldades para legitimar-se perante a sociedade e seus representantes devido ao subfinanciamento crônico, às fragilidades da gestão, à fragmentação de ações, ao corporativismo profissional, às desigualdades de ofertas e à articulação público-privada, entre outros aspectos (Lima, Silva, 2017). Dessa forma, evidenciam-se as dificuldades para alcançar os parâmetros de equidade, ou seja, prover acesso a tratamento e alocação de recursos a ricos e pobres, nacionais e migrantes, de acordo com as necessidades de cada grupo social.

Contemporaneamente, estudos evidenciam a complexidade do processo de integração de migrantes e pessoas refugiadas em países que os recebem (Jubilut, dez. 2018). A saúde, como direito essencial no contexto de integração, se destaca como um importante componente nesse processo (Granada et al., 2017; Santos, 2015; Sargent, Larchanché, 2011). Nesse sentido, é necessária uma abordagem interdisciplinar e intercultural focalizando a garantia dos direitos humanos no acesso à saúde e na integração dos migrantes. Para isso, é necessário considerar o modo como as pessoas concebem o adoecimento, como lidam com a saúde e a doença (Menendéz, 2016) e as intercorrências de adoecimento no processo migratório.

No contexto acadêmico, estudos realizados em diversas regiões do Brasil relacionam a migração e o acesso à saúde. Tais estudos trazem as experiências de migrantes e refugiados com o SUS, mostrando que esses enfrentam problemas de acesso diversos, tais como barreiras culturais e/ou linguísticas, racismo, xenofobia e preconceitos (Goldberg, Silveira, 2013; Silveira et al., 2013; Aguiar, Mota, 2014; Fernandes, Castro, 2014; Granada et al., 2017; Silva, 2017; Foster, 2017; Leão et al., 2017; Carneiro-Junior et al., 2018; Carvalho et al., 2018; Risson, Matsue, Lima, 2018; Haydu, 2017; Haydu et al., 2019). O racismo foi identificado em estudos com imigrantes e refugiados no país e em serviços de saúde mental (Carvalho et al., 2018; Risson, Matsue, Lima, 2018). O sofrimento associado ao refúgio, seja pelas condições pré ou pós-migratórias, pode ocasionar consequências para a saúde mental dessas pessoas (Galina et al., 2017).

Esses estudos apontam as dificuldades de inserção de migrantes e pessoas refugiadas no sistema de saúde em seus diversos níveis, evidenciando a complexidade dos processos migratórios transnacionais (Wimmer, Glick-Schiller, 2002; Silveira et al., 2013; Aguiar, Mota, 2014; Guerra, Ventura, 2017). Nesse sentido, faz-se necessário compreender como o contexto de pandemia, tão desfavorável àqueles que já sofrem as consequências do processo migratório (Rodrigues, Cavalcante, Faerstein, 2020), impactou as condições de vida e de saúde dessas pessoas.

A patologização da saúde dos migrantes tem sido trabalhada por Didier Fassin (2000), que alerta para os problemas relacionados com a associação feita entre a imigração e os processos de saúde e adoecimento. Segundo ele, já faz algum tempo que essa temática ocupa os manuais de saúde pública e de medicina tropical, em que podem ser encontradas três condições associadas à saúde e à imigração: a "patologia de importação", doenças parasitárias e hereditárias que os emigrantes carregam para os países de destino; as "patologias de aquisição", as novas condições ambientais encontradas pelo imigrante e que favorecem o desenvolvimento de doenças infecciosas e cardiovasculares; a "patologia de adaptação", as dificuldades de adaptação à sociedade de acolhimento, começando por problemas psíquicos que necessitam de atenção especial (Fassin, 2000, p.5). Essa lógica patologiza o corpo do migrante, naturaliza a associação desse corpo à doença e o penaliza duplamente como vetor e receptor de doenças. Sugere, o autor, que mais do que falar das doenças é necessário trabalhar no sentido de identificar quais são os desafios em termos de saúde e imigração que devem ser enfrentados pelas sociedades multiculturais. Portanto, é importante pensar nas complexas relações entre saúde, doença e sofrimentos decorrentes dos processos migratórios (Sargent, Larchanché, 2011; Pussetti, 2017).

Para Fassin (2001), o *status* ilegítimo de estrangeiros indocumentados está relacionado a uma percepção negativa de migrantes em geral. O racismo se torna uma base ideológica para restringir a legitimidade de movimentos transnacionais na França. Assim, o corpo de migrante também sofre com a ilegitimidade do corpo racializado.

Com relação a saúde e migrações, muitas vezes se torna problemática a associação da origem nacional com a suscetibilidade para o desenvolvimento de determinadas doenças ou, ainda mais complicado, tornar alguma nacionalidade vetor de uma doença.

Goldberg e Silveira (2013) mostram que não raramente o aumento do número de casos de tuberculose envolvendo imigrantes bolivianos na cidade de Buenos Aires, Argentina, a partir de 2004, era associado ao processo migratório como doença "importada". Profissionais de saúde de hospitais públicos indicavam o risco de bolivianos trazerem a doença do seu país e infectarem a população "nativa". A doença está relacionada ao contexto de vida (habitação, trabalho, alimentação etc.) que esses migrantes experimentam e sofrem no processo migratório. Estudos realizados por biólogos moleculares e especialistas mostraram que não existem provas científicas da transmissão de cepas nativas estrangeiras andinas a pacientes argentinos ("nativos") por imigrantes que chegam doentes à capital portenha ou contraem a doença; pelo contrário, a cepa dominante encontrada na Região Metropolitana de Buenos Aires começou a espalhar-se entre muitos dos imigrantes bolivianos que a haviam contraído. Interpretações preconceituosas acerca da doença e dos processos migratórios entre bolivianos em Buenos Aires dificultavam, aliás, o diagnóstico e a adesão ao tratamento da tuberculose.

## Considerações finais

Como todo trabalho realizado a diversas mãos, nossa proposta trouxe consigo os diferentes olhares das pesquisadoras e pesquisadores que constroem este texto coletivo, deixando transparecer ao longo dele as diferentes densidades da redação. As nossas conclusões apontam para a necessidade de aprimorar os dispositivos de acolhimento de imigrantes nos serviços de saúde, bem como a atenção e vigilância "antiexotista" da antropologia (Agier, 2001, p.9). Em relação à produção científica sobre saúde e migrações, porém, temos que ter atenção às diferentes áreas do conhecimento no sentido de não se estigmatizar qualquer nacionalidade por algum tipo de associação indevida a doença ou vírus ou mesmo a uma condição de maior suscetibilidade ao adoecimento que não seja em razão das condições de inserção nas sociedades nas quais os migrantes decidem deixar transcorrer suas vidas. A pesquisa sobre saúde e migrações no contexto pandêmico coloca em evidência os riscos de uma derivação xenofóbica e a necessidade de uma postura proativa no sentido de destacar os mecanismos perversos que podem vir a ocasionar preconceito e discriminações com relação a imigrantes.

Alguns estudos apontam também para a necessidade de se pensar a condição das mulheres migrantes, número que cresceu nas últimas décadas; um fenômeno global denominado de feminização (Castles, Miller, 2003) ou "genderização" das migrações (Yamanaka, Piper; 2006). Como mencionamos, as mulheres migrantes frequentemente representam corpos duplamente marginalizados e explorados, sendo necessário uma perspectiva interseccional para pensar a condição da mulher e de sua saúde.

Podemos sugerir, desse modo, que a pandemia teve como consequência colocar novamente em pauta a existência da xenofobia, o medo do estrangeiro, do fechamento de fronteiras, de exploração de gênero e de controle dos corpos, mas não de qualquer corpo, do corpo estrangeiro e indesejado, sempre suspeito de portar consigo uma doença maligna que pode vir a adoecer os nacionais, no caso em tela, de uma crise sanitária cujos impactos para os próximos anos ainda não podemos calcular com precisão; por isso convém estarmos atentos às derivas totalitárias, aos desejos de exclusão dos diferentes, vistos novamente como portadores de doenças em potencial, cuja presença colocaria em risco a vida daqueles que permanecem dentro das fronteiras do Estado-nação. Essa construção da "comunidade imaginada" do Estado nacional, para citar a formulação clássica de Anderson (1991), não é mais suficiente para nos dar a segurança prometida. A pandemia impôs uma nova "economia moral" (Fassin, 2009), e em um mundo em que livremente circulam vírus, micro-organismos e mercadorias, talvez a pandemia possa nos fazer repensar a circulação de seres humanos à luz de novas e desafiadoras propostas sobre nacionalidade e fronteiras.

AGRADECIMENTOS

Este texto é resultado parcial do projeto "Acesso à saúde e vulnerabilidades de migrantes internacionais no contexto de disseminação da covid-19: uma pesquisa interinstitucional em rede colaborativa", com apoio da Fapesp (processo 2021/06792-2), do CNPq (processo 403913/2021-7) e do projeto de pesquisa "A covid-19 no Brasil – fase 2: análise e resposta aos impactos sociais da pandemia – imunização, tratamento, práticas e ambientes de cuidado e de recuperação de afetados" (Ref. Finep 1212/21 – IAP-UFRGS 1862) que integra a Rede Covid-19 Humanidades. A pesquisadora coordenadora contou com financiamento resultado da Chamada CNPq/MCTI/FNDCT n.18/2021 – Faixa B – Grupos Consolidados. Processo 403913/2021-7 e Fapesp Roadmap Covid-19, processo 2021/06792-2.

## NOTA

[1] Aqui entendemos o chamado "distanciamento social" como o conjunto de medidas de redução das interações entre os indivíduos de uma comunidade, contemplando pessoas já e não isoladas. Essas medidas incluem o fechamento de escolas, locais de trabalho e comércio, proibição de eventos, redução e restrição da mobilidade inter e intramunicipal, entre outras, de acordo com Aquino et al. (2020).
